# Generators of Pressure-Evoked Currents in Vertebrate Outer Retinal Neurons

**DOI:** 10.3390/cells10061288

**Published:** 2021-05-22

**Authors:** Ji-Jie Pang, Fan Gao, Samuel M. Wu

**Affiliations:** Department of Ophthalmology, Baylor College of Medicine, Houston, TX 77030, USA; fgao@bcm.edu (F.G.); swu@bcm.edu (S.M.W.)

**Keywords:** mechanosensitive channel, TRPV, potassium channel, rod, temperature, patch clamping, immunofluorescence, confocal microscopy

## Abstract

(1) Background: High-tension glaucoma damages the peripheral vision dominated by rods. How mechanosensitive channels (MSCs) in the outer retina mediate pressure responses is unclear. (2) Methods: Immunocytochemistry, patch clamp, and channel fluorescence were used to study MSCs in salamander photoreceptors. (3) Results: Immunoreactivity of transient receptor potential channel vanilloid 4 (TRPV4) was revealed in the outer plexiform layer, K^+^ channel TRAAK in the photoreceptor outer segment (OS), and TRPV2 in some rod OS disks. Pressure on the rod inner segment evoked sustained currents of three components: (A) the inward current at <−50 mV (*I_pi_*), sensitive to Co^2+^; (B) leak outward current at ≥−80 mV (*I_po_*), sensitive to intracellular Cs^+^ and ruthenium red; and (C) cation current reversed at ~10 mV (*I_pc_*). Hypotonicity induced slow currents like *I_pc_*. Environmental pressure and light increased the FM 1-43-identified open MSCs in the OS membrane, while pressure on the OS with internal Cs^+^ closed a Ca^2+^-dependent current reversed at ~0 mV. Rod photocurrents were thermosensitive and affected by MSC blockers. (4) Conclusions: Rods possess depolarizing (TRPV) and hyperpolarizing (K^+^) MSCs, which mediate mutually compensating currents between −50 mV and 10 mV, serve as an electrical cushion to minimize the impact of ocular mechanical stress.

## 1. Introduction

The elevation of the intraocular pressure (IOP) is the most significant risk factor for glaucoma [1,2,3], including the mean level and fluctuation [4,5]. Glaucoma patients typically develop tunnel vision, but it is unclear why peripheral vision is more vulnerable to IOP elevation. Except for the sustained IOP level, retinal neurons are also exposed to IOP pulses, which are 2–3 times per second in primates [6], and the amplitude is enhanced under elevated IOP levels. The IOP in mice shows multiple peaks during the daytime [7]. Besides the pressure, glaucoma patients often show local retinal expansion in adults, known as optic disk cupping [8,9], and uniform retinal expansion in the case of buphthalmia in children. The IOP and retinal stretch, as well as the pulse of arteries and the fluctuation of local pressure, shape, and osmolarity, appear to be a suitable stimulus capable of opening mechanosensitive channels (MSCs) [10,11,12]. Despite the presence of retinal MSCs [13,14,15,16] and pressure-induced visual disorders [17,18,19,20], the role of MSCs in the outer retina under normal and pathological conditions is largely unclear.

Glaucoma studies often focus on inner retinal neurons, while these data have also indicated pathologies in outer retinal neurons. The retinal excitatory signal collected by retinal ganglion cells (RGCs) is initiated from photoreceptors and mediated by bipolar cells (BCs). In RGCs, our previous studies have shown reduced excitatory inputs upon the early stage of IOP elevation in glaucoma models [21,22], and others’ results support the damage by high IOP to the excitatory synapse from BCs to RGCs [23,24]. The electroretinography (ERG) b-wave, which is widely used to monitor the function of BCs, has shown some changes in both glaucoma patients [25] and animal models [26,27], and these functional changes, together with the pressure-related BC pathology [23,24] and pressure-sensitivity of BCs [16], support the involvement of the outer retinal neurons in glaucoma. However, the negative finding in BC populations in an earlier report [28] and the obvious pathology of RGCs demonstrate a lower structural vulnerability of outer retinal neurons in glaucoma. The damage to the rod ribbon synapse [29], rods, and the outer plexiform layer (OPL) [30] that has been revealed in glaucoma mouse models is yet to be explained. MSCs may mediate cation influxes capable of directly damaging the ultrastructure of cells [31,32,33], but the role of MSCs in photoreceptors in physiological and pathological conditions is still unclear.

Vertebrate retinal rods and the outer plexiform layer (OPL) have been reported to express two groups of MSCs, including the vanilloid transient receptor potential channel (TRPV) [34,35,36,37] and K^+^ channels such as the big potassium channel (BK, also known as the calcium- and voltage-gated large conductance potassium channel, Slo1, and KCNMA1) [38,39,40] and the two-pore domain K^+^ channel (K2P) TRAAK [41]. The K^+^ channels give rise to leak (also called background) K^+^ currents to stabilize the negative resting membrane potential and counterbalance membrane depolarization [42]. BK [43,44] and TRAAK are gated primarily by membrane tension [43,45,46,47,48,49]. TRPs belong to a superfamily of non-selective cation channels [11,12] more permeable to Ca^2+^ than Na^+^. TRPV1, TRPV2, and TRPV4 have been shown to open upon pressure, membrane stretch, hypotonicity, or fluid flow [10,11,50,51,52,53,54,55,56], with TRPV4 expressed in mammalian RGCs and the OPL [34,35] and TRPV2 in the OPL and axons of photoreceptors in the mouse [36], rat, cat, and primate retina [37]. K^+^ channels and TRPVs are activated by similar mechanical stimuli but mediate different currents. While studies often focus on the activity of individual channels, the combined effect has not been examined. We hypothesize that outer retinal neurons are responsive to pressure changes and the balance between MSCs reduces the impact of pressure-induced cation currents.

Antibodies have been widely used to identify MSCs, and the function of these channels may be quantitatively examined by patch-clamp recording techniques. In addition, FM1-43 has been used to reveal opened MSCs, because it becomes fluorescent only after entering the plasma membrane. It may quickly pass MSCs to label neurons in tens of seconds to a few minutes, and studies on hair cells from the inner ear have shown that the fast labeling of the cytoplasm is mediated by opened MSCs instead of endocytosis [57,58,59]. MSCs [58,59], including TRPV4 [57,60,61,62], contribute to the labeling in neurons. This study used immunocytochemistry, whole-cell patch-clamp recording, and FM 1-43 fluorescence and studied the expression and function of MSCs in the outer retinal neurons. The results demonstrated several MSCs in the outer retina, which mediate mutually compensating cation currents, serving to reduce the impact of pressure stress.

## 2. Materials and Methods

### 2.1. Animals

Laval tiger salamanders (Ambystoma tigrinum) were purchased from Charles D. Sullivan, Co. (Nashville, TN, USA) and KON’s Scientific Co. Inc. (Germantown, WI, USA) and handled as per policies on the treatment of laboratory animals of the Baylor College of Medicine and the National Institutes of Health. The animals were dark-adapted for 1–2 h before the experiment, and then, they were anesthetized in water containing MS222 until they were no longer responsive to touch or water vibration. The animals were then quickly decapitated, and the eyeballs were enucleated.

### 2.2. Single-Cell and Dual-Cell Patch-Clamp Recording of Photoreceptors

All procedures were performed under infrared (~1 mm) illumination with dual-unit Nitemare (BE Meyers, Redmond, WA, USA) infrared scopes. Whole-cell patch-clamp recording [63,64], preparation of living retinal slices [65,66], light simulation, immunofluorescence, and confocal microscopy [67,68] essentially followed procedures described in previous publications.

Axopatch 700A and 700B amplifiers were connected to DigiData 1322A interfaces and operated by pClamp software v9.2 and v10.3 (Axon Instruments, Foster City, CA, USA). Patch pipettes had a 5–8 MΩ tip resistance when filled with an internal solution containing 112 mM Cs-methanesulfonate, 12 mM CsCl, 5 mM EGTA, 0.5 mM CaCl_2_, 4 mM ATP, 0.3 mM GTP, 10 mM Tris, and 0.5% Lucifer yellow, adjusted to pH 7.3 with CsOH. For current-clamp and some voltage-clamp recordings, the pipettes were filled with internal solutions containing 112 mM K-gluconate, 10 mM KCl, 10 mM EGTA, 10 mM HEPES, 0.5 mM CaCl_2_, 1 mM MgCl_2_, 4 mM Na_2_-ATP, 0.3 mM Na_3_-GTP, and 0.5% Lucifer yellow, adjusted to pH 7.3 by KOH. A Cs^+^ -based internal solution is also a blocker for voltage-gated K^+^ channels and was used for differentiating K^+^ channel-mediated currents. The external Ringer’s solution contains 126 mM NaCl, 3 mM KCl, 3 mM CaCl_2_, 1 mM MgCl_2_, and 20 mM HEPES, adjusted to pH 7.3 with 10 M NaOH. The internal solution and the external normal Ringer’s solution yield an E_Cl_ of −59 mV at room temperature. Recorded cells were visualized by Lucifer yellow fluorescence with LSM 510 and LSM 800 confocal microscopes (Carl Zeiss, Oberkochem, Germany).

A photostimulator delivered light spots of a diameter of 600−1200 μm and 500 nm wavelength (λmax = 500 nm, full width at half maximum = 10 nm) at a series of intensities (−10 to −1 log I) to stimulate the retina via the epi-illuminator of the microscope [69,70,71]. Since we delivered uncollimated light beams through an objective lens of a large numerical aperture (Zeiss 40x/0.75 water), the incident light could enter the retina in many directions and, thus, had a minor photoreceptor self-screening effect [72]. The intensity of unattenuated (0 in log unit (log I)) 500 nm light from a halogen light source was 4.4 × 10^5^ photons μm^−2^ s^−1^.

Positive pressure (10–63 mmHg) steps were applied to cells during recording with a second patch pipette placed in the opposite direction to the recording pipette with a distance of ~100 µm. The pressure applied to the pipette was calibrated by a DM8215 digital manometer (Cole-Parmer, Vernon Hills, IL, USA) with a resolution of 0.57 mmHg [73]. In some well-studied mechano-gated channels [42], the convex membrane deformation facilitates the opening of mechanosensitive channels. The pharmacological channel modulators include TRPV4-specific agonists [34,35,74] 4α-phorbol 12,13-didecanoate (4αPDD, 2 µM), GSK1016790A (GSK, 2 µM), and RN1747 (20 µM; Tocris, Bristol, UK); TRPV4 antagonist RN1734 (5 µM); TRPV2 agonist 2-aminoethoxydiphenyl borate (2APB) [75,76]; BK channel blocker iberiotoxin (IBTX, 1 µM; Tocris); and general MSC blockers ruthenium red (RR, 25 µM) and Cd^2+^ (50 µM) [77,78,79]. Chemicals were purchased from Sigma-Aldrich (St. Louis, MO, USA) and Tocris Bioscience, except otherwise specified.

### 2.3. Multi-Cell Patch-Clamp Recording of Retinal Neurons 

Two EPC10 quadruplets (HEKA Instruments Inc, Holliston, MA, USA) were connected to provide 8 amplifiers for simultaneously recording 1–8 cells, and each of the channels could operate in either voltage- or current-clamp mode. The recording was performed under infrared illumination (>750 nm) or dim-red light and monitored by a Nano video camera (Stemmer Imaging AG, Puchheim, Germany). The light response of recorded neurons was evoked with a 505 nm LED driven by a digital output channel of the EPC amplifier. The size of the light spot was 700–1200 µm in diameter. Patch pipettes and the microscope were controlled by Luigs & Neumann SM-10 manipulators (Luigs & Neumann GMBH, Ratingen, Germany) with a resolution of 0.01 µm. The system had excellent stability [80,81] and allowed moving electrodes and changing the light intensity without vibrating the system. The light intensity was normalized with an optometer (United Detector Technology Inc., Hawthorne, CA, USA) for consistency among experiments.

### 2.4. FM 1-43 Probing for MSCs in the Open State

Light and environmental pressure were tested for whether to open MSCs permeable to FM 1-43. Living whole retinas or retinal slices were used. For testing the effect of light, the retinal tissue was set on a piece of cover glass and placed in a Petri dish, and a retinal area was further chosen under confocal microscopes (LSM 510 and LSM 800; Carl Zeiss, Oberkochen, Germany) with the dim transmission light. Then, the bath solution was replaced with 3 µM fixable FM1-43 for 30–60 s, during which time the local area was scanned with a 488 laser and several images were taken. Afterward, the tissue was washed several times and more images were taken. All images were taken under the same condition, including the pinhole size, laser intensity, gain, scanning speed, etc., and images from the selected region, which was simultaneously exposed to FM 1-43 and the 488 nm laser, were further compared with those from other regions to determine the effect of light on the labeling.

For testing the effect of environmental pressure, a pressure chamber was built by connecting a bottle to a stable air supply and a vent monitored with a digital manometer with a resolution of 0.57 mmHg (DM8215; Cole-Parmer, Vernon Hills, IL, USA) [73]. Retinal tissues of the experimental group were placed in the chamber and incubated in FM 1-43 for 60 s, and control tissues were handled similarly except in the open air. After several washes, all tissues were fixed and mounted for examination. Images of the experimental and control groups were taken under the same condition. The fluorescence was examined with a 488 nm argon laser and a 550–630 nm bandpass emission filter [82] with Zeiss confocal software. Fixable FM 1-43 (FM 1-43FX) was purchased from Invitrogen (F-35355; Waltham, MA, USA).

### 2.5. Antibodies and Immunocytochemistry

The preparation of vertical retinal sections and double- and triple-labeling followed our published experimental protocols [16,68,71,83,84,85]. Polyclonal rabbit anti-TRPV4 (LS-C135 [16], 1:200) was purchased from LifeSpan Biosciences, Inc. (Seattle, WA, USA). It was raised against rat TRPV4 (Q9ERZ8, aa853-871, peptide immunogen sequence: CDGHQQGYAPKWRAEDAPL). LS-C135 provided the best signal-to-noise ratio in the primate retina [16] and showed a similar specificity as LS-A8583 and LS-C94498 for labeling retinal TRPV4, which has been confirmed in TRPV4-knockout mice [34]. Polyclonal rabbit anti-TRPV2 (1:200, PC421; MilliporeSigma, Burlington, MA, USA) was produced with a synthetic peptide containing the amino acids 744–761 of rat TRPV2. It recognized the ~98 kDa (doublet) vanilloid receptor-like protein-1 in the rat spinal cord extract (vendor’s data), consistent with TRPV2 [75]. Goat anti-TRAAK (C-13) polyclonal antibody (1:100, sc-11324; Santa Cruz Biotechnology, Inc., Dallas, USA) was an affinity-purified goat polyclonal antibody raised against peptide mapping at the C-terminus of TRAAK of human origin. It recognized a single band of ~47 KDa (vendor’s data), consistent with TRAAK [86]. Two calbindin D-28k antibodies were used, one of which was a rabbit polyclonal antibody raised against the recombinant rat calbindin D-28k (CB) protein purchased from Swant (CH-1723, Marly 1, Switzerland) (CB38, 1:1000) [87,88], and the other was a mouse monoclonal antibody purchased from Sigma and produced with the bovine kidney calbindin-D (C9848, clone CB955, 1: 200) [87,88]. Other antibodies included goat calretinin antiserum (CG1, 1:1000, Swant [89,90]; AB149, 1:1000, MilliporeSigma) and polyclonal guinea pig anti-GABA (1:1K, AB175; Chemicon, Temecula, CA, USA) [91]. The staining pattern of the calbindin and calretinin antibodies was similar to previous studies, confirming the specificity.

Zeiss confocal microscopes (LSM 510 and LSM 800; Carl Zeiss, Oberkochen, Germany) and Zeiss imaging software were used for taking and analyzing images. Recorded cells were visualized by Lucifer yellow fluorescence or/and neurobiotin labeling. A series of optical sections were often made over a targeted cell or region. The airy scan, as well as the regular line and frame scan, were used for some morphological studies, which could provide the best confocal resolution (30 nm per pixel and a step of 150–180 nm).

### 2.6. Statistics

Data were analyzed with Sigmaplot v11.0 (Systat, Point Richmond, CA, USA), Clampfit v9.2 and v10.3 (Axon Instruments, Foster City, CA, USA), and Microsoft Excel v1708 (Microsoft Co., Redmond, WA, USA) and presented as the mean ± s.e.m. Regression analysis and Student’s *t*-test were performed, and the two-tailed *p*-value was reported in all cases. The peak amplitude of the responses (*R*) of photoreceptors to pressure stimuli (*P*) was well fit to an exponential rise to the maximum function *f(P) = R_max_ (1-e^((−b/P)).* The data collection was completed before data analysis and was independent of data interpretation. The α level for rejecting the null hypothesis was 0.05.

## 3. Results

### 3.1. The Immunoreactivities of the Mechanosensitive Potassium and Non-Selective Cation Channel in Outer Retinal Neurons

**Figure 1:**
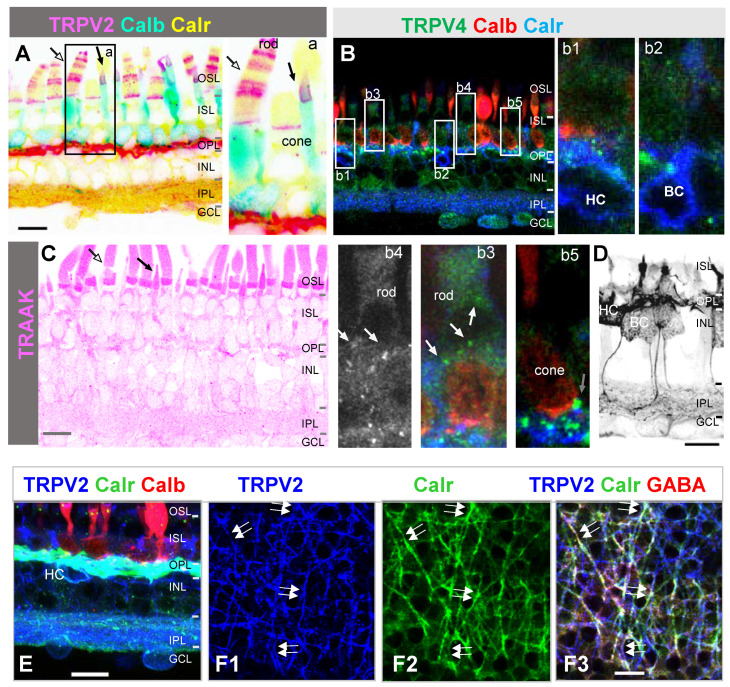
The expression of several MSCs in the salamander retina. Confocal images show the retina slice triple-labeled for calbindin D-28k (Calb), calretinin (Calr), and TRPV2 (**A**,**E**,**F**) or TRPV4 (**B**) or probed for TRAAK (**C**). (**A**) TRPV2 antibody brightly labels the outer plexiform layer (OPL), half disks in the rod outer segment (OS) (white arrow), and cone OS (black arrow) and weakly reveals some processes in the inner plexiform layer (IPL) and somas in the ganglion cell layer (GCL). (**B**) Bright TRPV4-immunoreactive puncta are mostly present in the OPL and terminals of cones (black arrow, (**b5**)), while weaker TRPV4 signals are visible in somas and dendrites of horizontal cells (HCs, (**b1**)), bipolar cells (BCs) (**b2**), the basal membrane of rods and putative rod axon terminals (white arrow, (**b3**,**b4**)), the inner plexiform layer (IPL), and somas in the GCL. (**C**) TRAAK antibody labeled the OS of single cones (black arrow) the most brightly, and it clearly revealed the OS of rods (white arrow). Weaker TRAAK signals are sparsely present in the OPL and IPL. (**D**) Calretinin antibody heavily labeled HCs and clearly labeled some BCs. (**E**) Calretinin-labeled soma of HCs positive for TRPV2. (**F**) At the OPL focal plane, nearly half dendrites of HCs that are identified by calretinin (**F2**) and GABA ((**F3**), red) are labeled for TRPV2 (**F1**) (white double-arrow). (**F1**,**F2**) display the blue and green channels of (**F3**), respectively. OSL: outer segment layer; ISL: inner segment layer; RGC: retinal ganglion cell. Scale bars: 20 µm.

We first examined the immunoreactivity of several MSCs in the retina. Rods and cones were differentiated by the shape of the outer segment (OS). Calbindin D-28k (Calb) antibody was used to label the OS, soma, and axon terminal (Figure 1) [92,93] of single and double cones. Calretinin (Calr) and GABA antibodies brightly labeled the soma and processes of horizontal cells [90] (Figure 1D), and calretinin also stained some ON-bipolar cells on the soma, Landoit’s club, dendrites, and axons (Figure 1A,B,D). In retinas triple-labeled for TRPV2, calbindin, and calretinin, TRPV2 signals were primarily found in the outer retina (Figure 1A), which brightly revealed half disks in the rod OS, cone OS, and the outer plexiform layer (OPL), including half dendrites of horizontal cells (HCs) identified by calretinin and GABA antibodies [91] (Figure 1E,F). One OS of double cones was labeled brighter, while the cytoplasmic membrane of the rod OS was negative for TRPV2.

In retinas triple-labeled for TRPV4, calbindin, and calretinin, TRPV4 immunoreactivity appeared as large and fine puncta, and the former was primarily in the OPL and the latter in the terminals of cones and rods, the IPL, and somas in the ganglion cell layer (GCL) (Figure 1B). The immunoreactivity of TRAAK was primarily present in the OS of photoreceptors, and it brightly revealed the OS of single cones and clearly labeled the rod OS. Some smaller puncta were present in the OPL and IPL. These data demonstrate that each of the MSCs has a unique distribution pattern, and their proportion varies among the neurons and cellular compartments with the rod OS disks, rod OS membrane, cone OS, OPL, and the axon terminals of photoreceptors, IPL, and GCL expressing TRPV2, TRAAK, TRPV2-TRAAK, TRPV2-TRPV4-TRAAK, and TRPV2-TRPV4, respectively.

### 3.2. Pressure-Evoked Currents in Rods

**Figure 2:**
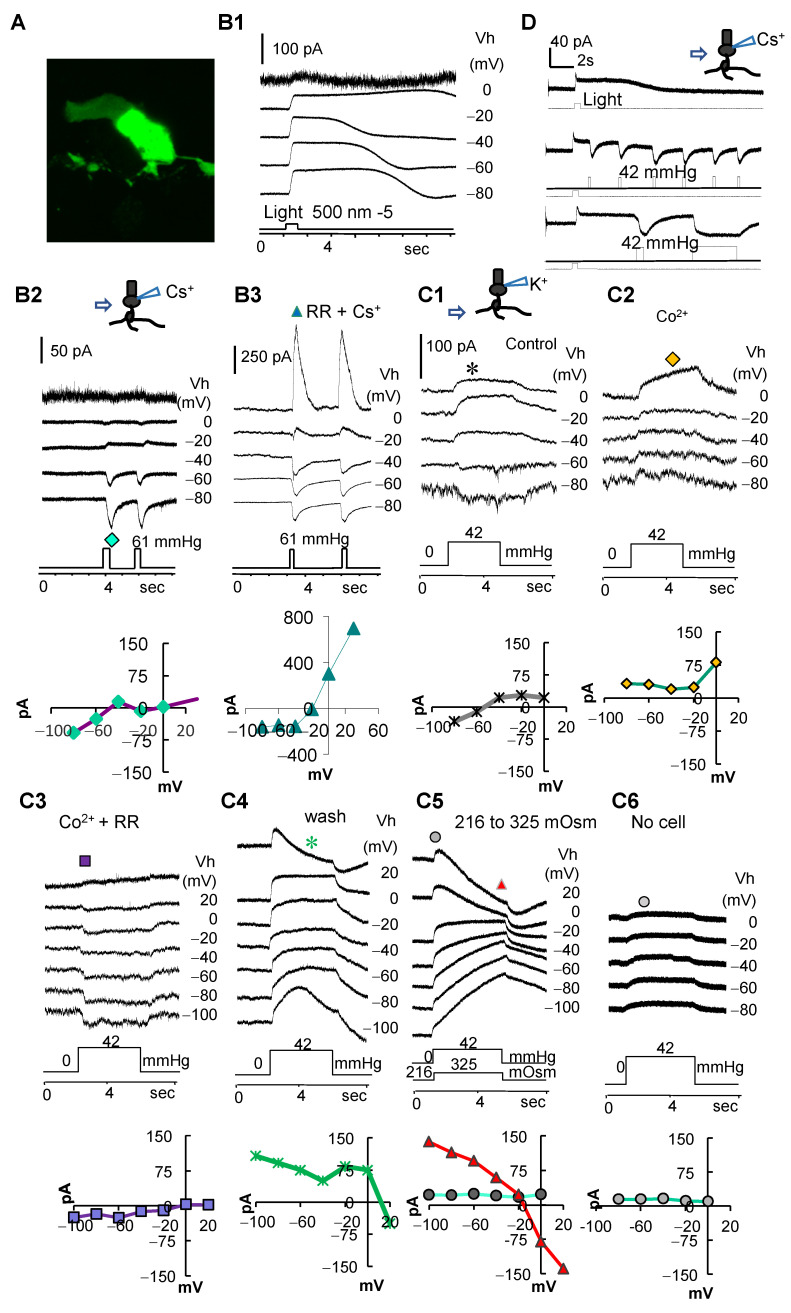
Pressure-evoked currents in rods. Rods are recorded in voltage-clamp mode with a patch pipette filled with an internal solution containing Cs^+^ 137 mM (**B**) or K^+^ 132 mM (**C**) and labeled with Lucifer yellow (**A**). (**B**) A rod responds to light steps with outward currents (**B1**) at different holding potentials (Vh), and the short dynamic pressure pulses applied with a puffer pipette to the soma of the rod elicited primarily inward currents (*I_pi_*) (**B2**). Ruthenium red (RR) shifted the reversal potential from −50 mV to ~ −20 mV and unmasked a non-selective cation conductance (*I_pc_*) (**B3**). (**C1**) Longer pressure steps were applied to the outer plexiform layer and evoked currents reversed around −50 mV. (**C2**) In the presence of synaptic blocker Co^2+^, the evoked current was primarily outward (*I_po_*). (**C3**) In the presence of Co^2+^ and RR, the evoked current reversed at ~10 mV, unmasking a non-selective cation conductance (*I_pc_*). (**C4**) The outward current was washed back. (**C5**) In a hypotonic bath solution, a hypertonic puffer solution was applied with a dynamic pressure step and induced a slow decrease in the non-selective cation conductance (triangle), which was reversed around −20 mV and superimposed on an outward current (dot). (**C6**) The same dynamic pressure from the same puffer pipette caused a rather small outward current on a clean patch pipette placed at the same distance. (**D**) The response of a rod to both light and pressure recorded with a Cs^+^-containing internal solution. The results demonstrate three major components of the pressure-evoked current in rods.

To determine the function of MSCs in the retina, we first examined photoreceptors for the response to the mechanical and osmotic pressure (Figure 2). Rods (*n* = 9) are recorded with a patch pipette containing a Cs^+^- (Figure 2B) or a K^+^-based (Figure 2C) internal solution, and recorded cells were labeled with Lucifer yellow. In healthy rods that could generate normal light responses, the dynamic pressure applied to rod soma and the OPL with a patch pipette and the osmotic pressure focally applied directly to rods with a pipette or in the bath all evoked sustained responses in rods, demonstrating the mechanical responsiveness of rods. We further used the reverse potential of the pressure-evoked current, its dependence on K^+^, and the effect of synaptic blocker Co^2+^ and a general MSC blocker ruthenium red (RR) to determine the type and location of MSCs involved (*n* = 18) (Figure 2).

First, when a K^+^ internal solution was used (Figure 2C), pressure steps applied to the OPL evoked a current reversed around −50 mV, and Co^2+^ blocked the inward current component (*I_pi_*) at <−50 mV, and the residue current was purely outward (*I_po_*) at ≥−80 mV. In the presence of Co^2+^ and RR, the evoked current reversed at ~10 mV, revealing another component, a non-selective cation conductance (*I_pc_*). The results indicate that the pressure-evoked current consists of three components: a chemical synapse-mediated inward current, an outward leak current, and a non-selective cation current. Second, when a Cs^+^-based internal solution was used to block some potassium channels (Figure 2B), applying dynamic pressure to the soma of rods elicited mainly inward currents with negligible outward currents and a reversal potential near −50 mV, supporting that *I_po_* is mediated by mechanical sensitive K^+^ channels. Ruthenium red (RR) with both K^+^- and Cs^+^-based internal solutions shifted the reversal potential of the evoked current from ~ −50 mV to ≥−20 mV, consistently revealing a non-selective cation current. Third, osmotic pressure applied with a hypertonic puffer solution (Figure 2C5) induced a slow decrease in the non-selective cation conductance, and its late phase reversed around −20 mV. This, consistent with the morphological data, indicates that TRPV4 is present in rods. The pressure–response curve of rods was well fit to an exponential function f(P) = 16.66 (1−e^ ([−4.45P])) (two-tail *p* < 0.002) and saturated at ~0.5 psi (25.9 mmHg) at the membrane potential level (−40 mV). To estimate the physical artifact of the puffer solution on the recording pipette, we compared the response of a recording tip with and without a cell attached, and we found that the artifact was a rather small sustained outward current without a reversal potential (Figure 2C6). The data together indicate that rods are responsive to pressure and osmotic changes, and the pressure-evoked currents *I_pi_*, *I_po_*, and *I_pc_* are mediated by presynaptic neurons, mechanosensitive K channels in rods, and mechanosensitive TRPVs in rods, respectively.

**Figure 3:**
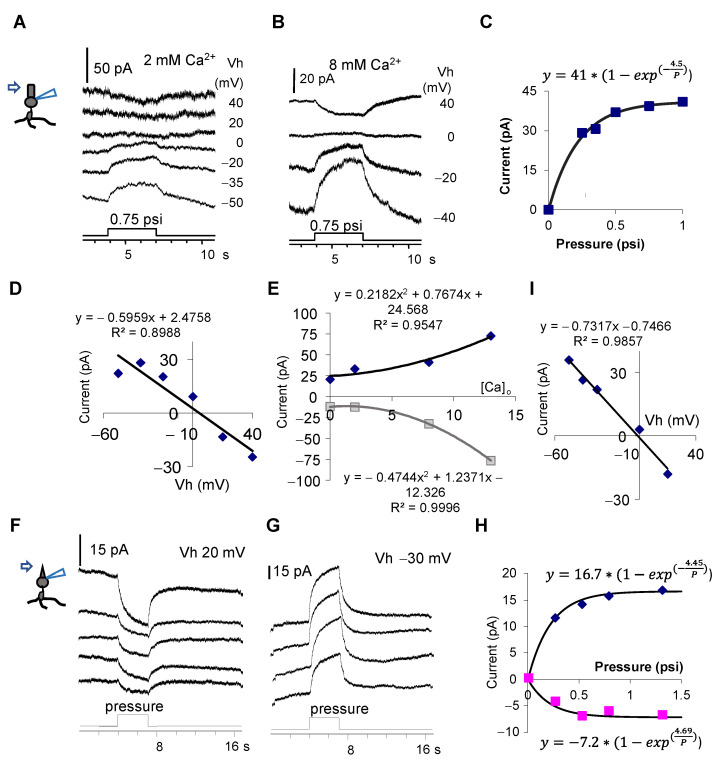
Response of photoreceptors to pressure applied to the outer segment. (**A**,**B**,**F**,**G**) depict the pressure-evoked current. With the internal Cs^+^ blockage of K^+^ channels, the pressure causes a sustained decrease in the cation conductance in a rod (**A**,**B**), which reverses at ~5 mV ((**D**), at 0.5 psi) and saturates at ~0.5 psi (**C**). (**E**) The amplitude of the pressure-evoked current at −30 mV (diamond) and 20 mV (square) increases upon increasing the extracellular Ca^2+^ concentration ([Ca^2+^]_o_) (two-tailed *p* = 0.0229 and 0.0002, respectively). The pressure–current response curve was well fit to an exponential rise to the maximum function. The two-tailed *p* for *R_max_* is 0.0001, and *p* for *b* is 0.0004. F and G show the pressure-evoked current in a cone at −30 mV (**G**) and 20 mV (**F**), which reverses near 0 mV (**I**), and the pressure–current response curve (**H**) was well fit to two exponential functions. For the upper equation (−30 mV), the two-tailed *p* for *R_max_* and *b* is 0.0001 and 0.0020, respectively. For the lower equation (20 mV), the two-tailed *p* for *R_max_* and *b* is 0.0080 and 0.0442, respectively. The data indicate that the extracellular pressure applied to the outer segment causes the closure of TRPV2 in the outer segment of photoreceptors.

To understand the role of TRPV2 in the outer segment (OS), we further focally applied pressure to the OS of rods and cones. In the presence of internal Cs^+^, both rods (Figure 3A–E) and cones (Figure 3F–I) showed the pressure-evoked closure of a non-selective cation current reversed at −5 to 0 mV (Figure 3D,I). The response was enhanced by the increase of the extracellular Ca^2+^ concentration [Ca^2+^]_o_ (Figure 3A,B,E). The pressure response was saturated around 0.5 psi, with a τ value of ~0.22 psi (τ = 1/b) (Figure 3C,H). The data, together with those in Figure 1, indicate that in the presence of internal Cs^+^, the pressure on the outer segment of photoreceptors closes the cation conductance in line with TRPV2.

### 3.3. The Effect of the Pharmacological and Thermal Modulation of Mechanical Sensitive Channels on the Light Response of Rods

**Figure 4:**
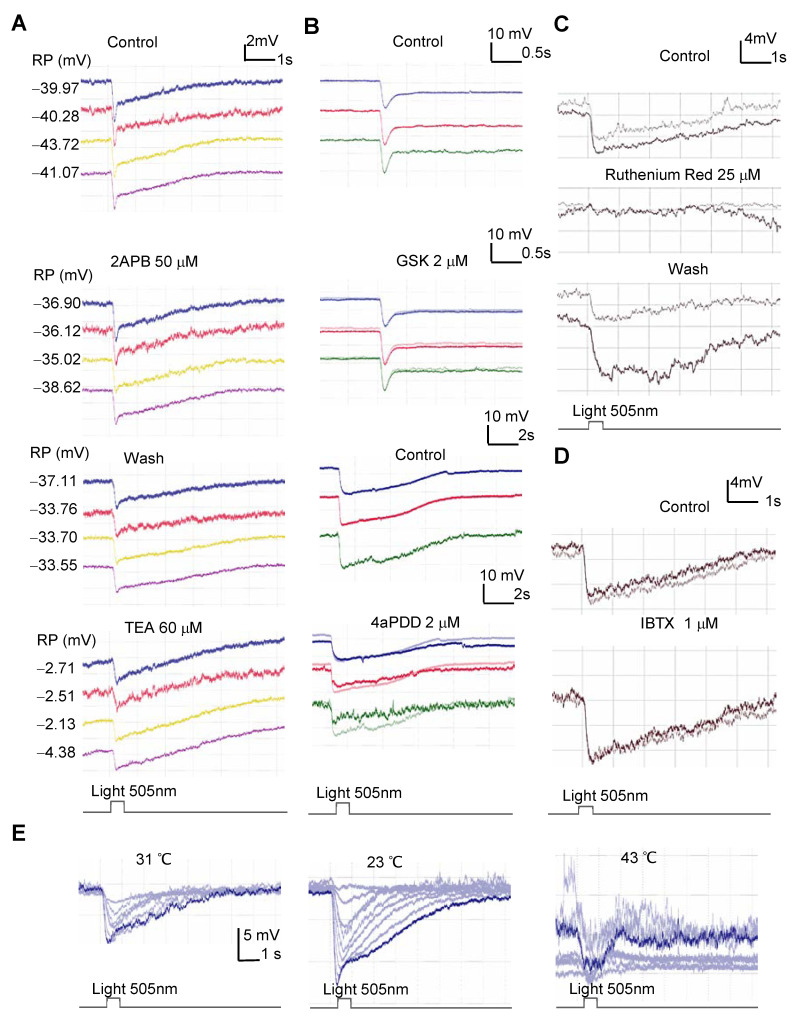
The effect of modulators of the mechanical sensitive ion channels on the light response of rods. Rods were recorded for the light response in current-clamp mode. (**A**,**B**) Three to four rods were simultaneously recorded with the multi-cell patch-clamp technique. TRPV2 agonist 2APB weakly depolarizes rods and slightly reduces the light response (**A**). The K^+^ channel blocker TEA largely depolarizes rods and reduces the light response. (**B**) TRPV4 channel agonists GSK and 4aPDD slightly hyperpolarize rods (the highlighted trace). (**C**) The light response in a rod was largely and reversibly reduced by ruthenium red (RR). (**D**) A blocker for BK channels, IBTX, enhances the light response of a rod. The two traces in each panel in C and D were recorded 2–3 min apart. (**E**) The light-evoked potentials recorded at eight light intensities are larger at 23 °C than at 31 °C and severely disrupted at 43 °C. (**B**–**E**) Membrane potentials were maintained at ~−40 mV.

Then, we examined the effect of the modulation of MSCs on the light response of rods (Figure 4). The TRPV2 agonist 2APB depolarized the resting potential (RP) by 10.44% (3.07–8.7 mV, *p* = 0.001, *n* = 7) and reduced the amplitude of the light responses by 13.29% (two-tail *p* = 0.037, *n* = 7). The MSC blocker RR decreased the amplitude of the light response in some rods (−42.84%, two-tailed *p* = 0.134, *n* = 4), while the changes in the RP also varied among the cells (*p* = 0.473). TRPV4 agonists GSK and 4αPDD weakly hyperpolarized the RP by 4.97% (0.38–3.7 mV, two-tailed *p* = 0.02, *n* = 6) without significantly reducing the light response (−20%, *p* = 0.113). TEA is a blocker for most potassium channels. It largely depolarized rods (32.85 mV, two-tailed *p* < 0.0001, *n* = 4) and reduced the light response by 23.72% (two-tailed *p* = 0.007, *n* = 4). The BK channel blocker IBTX enhanced the amplitude of the light response of some rods. The data demonstrate that modulating retinal MSCs could variably affect the RP and light response of rods, while the overall effect of the TRPV and K channels are generally mutually antagonizing.

Most MSCs are temperature sensitive. Here, we tested the effect of a temperature change on the light response of rods. At room temperature (~22 °C), rods showed the largest amplitude of light-evoked potentials at the resting potential level. The amplitude reduced when the temperature was raised to 31–33 °C, and a temperature of 40–44 °C severely disrupted the light response and transiently enhanced the baseline noise. The latter effect was not reversible. This result is in line with our previous observations. In the past more than 20 years, our laboratory has used salamander retinas for vision research, and we noticed that the retinal function and structure were often less healthy in summer compared with those in winter, including the less organized tissue structure, the “spotty” look of somas, and the difficulty to record larger light responses. These data together demonstrate the thermosensitivity of visual signals, further supporting the presence of MSCs in rods.

### 3.4. FM 1-43-Identified Open Channels in the Outer Segment (OS) of Photoreceptors

**Figure 5:**
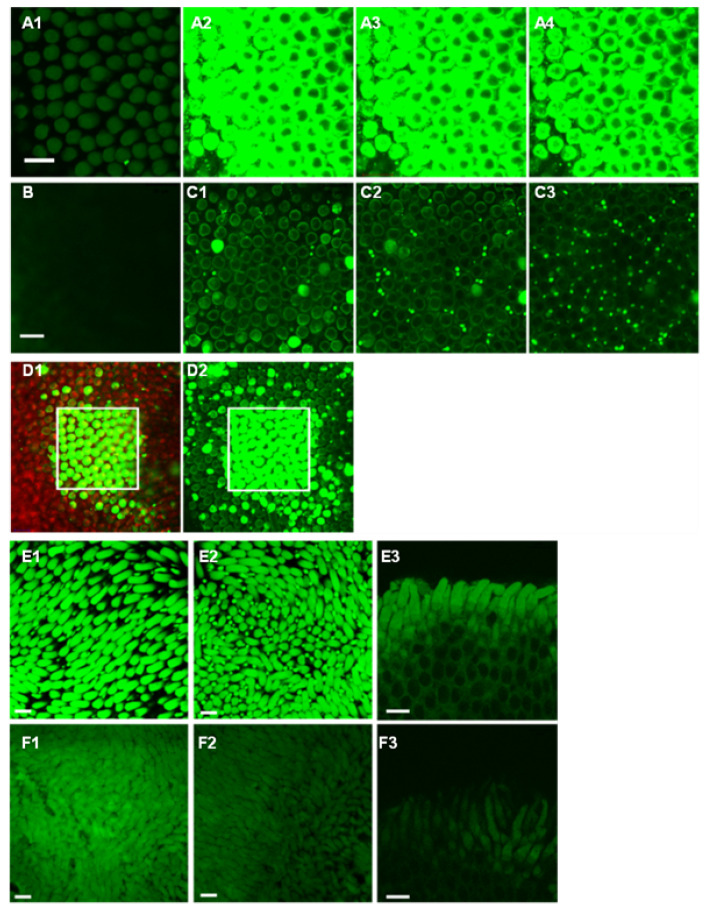
The modification of light (**A**–**D**) and pressure (**E**,**F**) (28 mmHg, (**E**)) on the FM 1-43-labeled open channels. (**A**) A selected area during the incubation of FM 1-43 at the focal plane of the outer segment (OS). (**A1**–**A4**) were taken from 0 s, 20 s, 50 s (begins wash), and 2 min after applying the drug, respectively (**B**) The OPL of the selected area after 20 min. (**C**) Other areas at the OS plane after 49 min, showing a low density of labeled rods (**C1**) and single and double cones (**C2**,**C3**). Labeled double cones are less restricted to the selected region. All images in A to C were taken under the same conditions. (**D**) A large region including the selected area (white square) after 66 min. The green channel in (**D1**,**D2**) shows FM 1-43, and the red channel in D1 is the Nomarski image. The data indicate that the 488 nm laser illumination opened some channels permeable to FM 1-43. (**E**) A piece of the flat-mount retina was simultaneously exposed to 3 µM FM 1-43 plus 28 mmHg for 80 s compared with the other piece from the same eye that was exposed to the same drug without the pressure (**F**). (**E1**–**E3**) and (**F1**–**F3**) are images from different locations. (**E3**,**F3**) were taken from the edge of the tissue. The fluorescence is the brightest in the outer segment and enhanced by the pressure. All images in (**E**,**F**) were taken under the same conditions. Scale bars: 20 µm. The data indicate that light and pressure open channels permeable to FM 1-43.

Furthermore, we investigated the role of MSCs in intact photoreceptors (Figure 5) with FM 1-43 fluorescence. First, we examined how the amount of FM 1-43-labeled open MSCs were related to light. In flat-mount retinas (Figure 5A–D) (*n* = 5) or slice preparations (*n* = 7), a retinal region was selected and focused under dim transmission light. Then, 3 µM FM 1-43 was applied to the bath for 50 s, during which time only the selected region was scanned by a 488 nm laser and imaged several times. After washing away FM 1-43, more images were taken from the selected area and compared with images from the surrounding areas. The results showed that the selected area exhibited a nearly perfect square shape clearly distinguishable from the surrounding region after 20 s, indicating that the light enhances the channel opening. From 50 s to 2 min, all OSs of photoreceptors in the selected area were brightly fluorescent, while labeled OSs in other regions were rather sparse (Figure 5C). At 49 min, more OSs of double cones in surrounding regions (Figure 5C2,C3) were labeled than those of rods. The data indicate that rods and cones possess FM 1-43-permeable channels, which may be opened by light and closed in darkness. The 488 nm laser scanning was normal and brief. The effects of light onset and offset were not distinguished.

Second, we examined how pressure affects the amount of the FM 1-43-labeled open MSCs in photoreceptors (*n* = 5) (Figure 5E,F). In the retina exposed simultaneously to 3 µM FM 1-43 in the bath and an environmental pressure of 28 mmHg for 80 s, the fluorescence in the OS of photoreceptors was much brighter than that in the control retina treated with 3 µM FM 1-43 in the open air for 80 s. It indicates that the pressure opens MSCs, allowing FM 1-43 to pass. Notably, FM 1-43 fluorescent signals mostly highlighted the somatic membrane instead of the disk membrane of rods (Figure 5A), indicating that the labeling of FM 1-43 probably does not involve TRPV2 in the disk of the rod OS. Although a 488 nm laser is not fully comparable with normal light signals, these data demonstrate that the pressure-enhanced labeling of the FM 1-43-permeable channels is comparable to the light-induced labeling, and the channel opening is likely physiological and could affect the photocascade and generate visual noise.

## 4. Discussion

### 4.1. Rods and Presynaptic Neurons Express Mechanical Sensitive Channels

MSCs have been reported in the OPL and photoreceptors (Table 1), and previous studies have shown some glaucoma-induced damage to rod ribbons [29,30], BC inputs, and BC synapses in RGCs [21,22,23,24]. However, how MSCs in outer retinal neurons mediate pressure responses under normal and glaucomatous conditions is not clear. Consistent with previous observations [16], this study revealed immunoreactivities of several types of MSCs in the OPL and photoreceptors. Our data further showed that photoreceptors respond to pressure with sustained currents mediated by two families of MSCs, and the results demonstrate that the vertebrate outer retina expresses functional MSCs and probably contributes to the glaucomatous pathophysiology in photoreceptors and BCs.

Horizontal cells (HCs) have not been previously reported to be mechanoresponsive. Our data showed that the pressure-evoked current consisted of a Co^2+^-sensitive inward current component at <−50 mV (*I_pi_*) and a Co^2+^-insensitive component, which indicates that rods and some presynaptic cells both possess MSCs. Meanwhile, our immunological results revealed strong TRPV2 signals in the OPL. Some calretinin/GABA-positive HCs [91] expressed TRPV2 (Figure 1), which may explain *I_pi_* if assuming that TRPV2 in the OPL is also closed by pressure as in the rod OS (Figure 3; also see below) and a negative feedback synapse is formed from HCs to rods [90,94,95,96]. Further investigation is required to determine the cell types possessing TRPV2 signals in the OPL, in addition to GABAergic HCs [92,93,94,95,96,97]. TRPV4 is expressed in primate bipolar cells [16], and data from this study (Figure 1B) are generally consistent with the previous report.

### 4.2. Pressure-Evoked Currents in Rods Are Compartmental and Involve at Least Three Generators

After blocking *I_pi_*, pressure on the inner segment of rods activated an outward leak conductance (*I_po_*) at ≥−80 mV accountable by K^+^ channels such as BK and TRAAKs and a cation conductance reversed around 10 mV (*I_pc_*) partially accountable by TRPV4 and TRPV2. Vertebrate photoreceptors have been reported to express BK [38,39,40], TRAAK [41], and TRPV2 [36,37], and the OPL expresses TRPV4 [16,34,35]. The co-expression, function, and interaction of these channels have not been clarified before. Our data, consistent with these reports, demonstrate that pressure may simultaneously open the two families of channels, which mediate mutually compensating currents in the same rod. BK is sensitive to internal Cs^+^ blockage [99], while TRAAK is less sensitive [100,101]. However, considering the presence of TRAAK in the OS, we thought that the contribution of TRAAK to *I_po_* could hardly be fully excluded.

Interestingly, when pressure was applied to the outer segment, it closed the cation conductance reversed around 0 mV. Meanwhile, in contrast to the previous study, we observed TRPV2 in some disks of photoreceptors and TRPV4 in the OPL. The data together demonstrate that the outer and inner segments of rods do not uniformly express TRPV2 and TRPV4. Pressure-induced cation currents from the inner and outer segments showed an opposite polarity, and this mechanism likely critically serves to reduce the impact of the pressure on rods.

### 4.3. Pressure-Evoked Mutually Compensating Currents in Rods

As mentioned above, the currents evoked by pressure on the inner segment (*I_pc_*) and the outer segment are mutually compensating. The former is the opening of a cation conductance with a reversal potential of 10 mV, while the latter is the closure of a cation conductance with a reversal potential of ~0 mV accountable by TRPV2. TRPV2′s closure is accountable by its location, which is on the disk of the OS rather than the cytoplasmic membrane.

The currents evoked by pressure on the inner or outer segment itself are also mutually compensating. First, *I_pi_* is the inward current at <−50 mV, *I_po_* is the outward current at ≥−80 mV, and *I_pc_* is the inward current at <10 mV. This mechanism is anticipated to effectively reduce the mechanoelectrical noise in rods. Second, the environmental pressure caused the opening of some channels in the OS (Figure 5), while TRPV2 appeared to be closed. The former is likely to involve the opening of BK and TRAAK channels (Figure 1 and Figure 4, and Table 1), serving to antagonize the effect of TRPV2. In our results, FM 1-43 signals were the brightest in the cytoplasmic membrane of rods, and TRAAK (Figure 1) [40] and BK [38,39,40] were not found in the disk of the OS; thus, FM 1-43 presumably enters the cells via these K^+^ channels on the cytoplasmic membrane of the OS.

Rods respond to extracellular hypertonicity with the closure of cation currents resembling *I_pc_* except for the opposite polarity, supporting the expression of TRPV4 [102,103]. TRPV4 has been shown to be sensitive to RR, while our *I_pc_* is a residue component after RR blockage. RR is a general blocker for MSCs, and whether TRPV4 in this species has a unique structure is unclear. TRPV4 is also opened at a warm temperature of around 27 °C [104,105]. In our results, photocurrents in rods were reduced by the opening of TRPV4 with the specific agonist 4αPDD and heating from 23 to 31 °C (Figure 4). The data together support that TRPV4 is present in rods and mediates pressure-evoked cation currents.

Neurons are required to cope with mechanical stress like other eukaryotic cells. Our data together indicate that for normal rods at −50 to 10 mV, mechanosensitive K^+^ channels and mechanosensitive TRPVs mediate mutually compensating mechanoelectrical currents, which may serve as an electrical cushion to minimize the impact of mechanical disturbance on visual signals. The compensation is present between the presynaptic and postsynaptic neurons and between the inner and outer segments of rods; thus, any unbalanced expression of the two families of MSCs and damage to the synapses are anticipated to cause pathological responsiveness of neurons to mechanical stresses.

Multiple MSCs are expressed in the central nervous system of mammals. Since pressure and osmolarity may fluctuate under physiological and pathological conditions, we propose that the compensation or balance between the two families of MSCs in neural networks is important for performing normal neuronal functions, and the mechanism is likely reserved for vertebrates, including mammals. Whether losing such balance involves the pathogenesis of glaucoma is worthy of further research.

### 4.4. Mechanosensitive Channels Involve the Maintenance of the RP, While TRPV2 Probably Also Involves Phototransduction

BK, TRAAK, and TRPVs are known to mediate cation currents. How they affect the function of rods is not clear. K^+^-permeable MSCs often mediate leak/sustained currents, while our data showed that *I_po_*, *I_pi_*, and *I_pc_* of rods are all sustained. These data, consistent with our pharmacological studies (Figure 4), indicate that changes in the environmental pressure may modify the resting membrane potential (RP) of photoreceptors to affect neurotransmitter release. Notably, in our results, modulators of MSCs, except for RR and TEA, could only weakly modify the RP and photocurrents of rods, which is likely due to the mutually compensating effects of the same MSCs located at different neurons or compartments.

TRPs are used to detect light signals in drosophila photoreceptors [106], and TRPM1 has been found to mediate the light response in mammalian ON-bipolar cells [107,108]. However, TRPs in the vertebrate retina have not been reported to involve the phototransduction cascade in photoreceptors. Mammalian glaucoma models have showed damages to the ribbon synapses of rods and the OPL [29,30], and the mechanism is not well understood. We observed clear TRPV2 signals in rod discs, heating from 33 to 44 °C severely disrupted the light response of some rods, and RR showed profound reversible suppression on the light response of some rods. These data suggest that the normal function of TRPV2 is probably required for the phototransduction cascade in vertebrate rods.

### 4.5. Rigid Shape of the OS of Normal Rods Likely Helps to Open TRPV2 in Rod Disks

TRPV2 has been reported in photoreceptor axons and the OPL in the mouse [36], rat, cat, and primate retina [37]. It is also expressed in immune cells, mediates the phagocytosis of the retinal pigmental epithelium (RPE), and plays an important role in photoreceptor regeneration related to macular degeneration [109,110]. However, the functional significance of TRPV2 in retinal neurons is largely unknown. Like previous studies, our morphological data revealed the expression of TRPV2 in the OPL and axons of photoreceptors. In addition, we observed TRPV2 in some disks of the rod OS.

Our results revealed that in the presence of internal Cs^+^, applying pressure perpendicular to the OS could close the cation conductance in rods, and this appears to indicate that normal TRPV2 is in an open state and pressure closes TRPV2. The rigid cylinder shape of the rod OS seems to be capable of opening TRPV2 if the disk membrane where TRPV2 resides is extended by attaching to the cytoplasmic membrane. The attachment has been, indeed, observed [111,112], though rod disks have often been described as closed structures. In biochemical studies, wild-type rat TRPV2 has been reported to be fully open [113], and the spatial structure of the agonist-free full-length TRPV2 molecule [75] shows larger upper and lower gates compared with agonist-opened TRPV1. The channel opening is thought to involve the location of some lipid molecules [75,114]. TRPV2 is sensitive to mechanical stresses and other factors [56,76,115,116], though it has a temperature threshold of 53 °C [115,117]. In our results, the TRPV2-immunoreactive signal in rods (Figure 1) is nearly perfectly horizontal, flat, and perpendicular to the vertical axis of rods, supporting a positive tension presented along the disk surface. Meanwhile, pressure primarily enhances the FM 1-43 signal near the cytoplasmic membrane of the OS. These data together support the idea that TRPV2 in the disks of the rod OS may open under physiological conditions and close upon pressure due to the location, while the mechanical sensitive K^+^ channels in the cytoplasmic membrane may open upon pressure like typical MSCs [11,42,49,118].

Mammalian retinal cones have a rigid shape like salamander rods [92], and our recent data have shown that the PNA shell of mammalian cones has unique and variable structures (not published). While central vision is less vulnerable in glaucoma, whether the rigid shell structure of cones is protective against pressure-induced damage is still unknown.

Aquatic animals have a much higher opportunity to experience changes in the outer ocular pressure than humans. The rigid shape of the OS of rods and cones in the salamander retina could probably improve the pressure tolerance of photoreceptors, and the opened TRPV2 appears to be able to compensate the pressure-induced *I_pi_* and *I_pc_* in the soma and outer plexiform layer, adding an extra layer of protection.

This study does not include all the MSCs found in the outer retina. The rod IS also expresses Piezo1 and TRP canonical 1 (TRPC1) [119,120]. Piezo1 in the dorsal root ganglion neurons mediates rapidly adapting mechanically induced cation currents, which reverse around 10 mV [121,122]. TRPV4 has been known as an osmotic sensor [103,122,123,124,125], and hypotonicity evoked clear cation currents in rods in our results, which support the expression of TRPV4 in rods. However, TRPV4 immunoreactivity in rod somas and putative axonal terminals are weak; thus, our data themselves could not exclude *I_pc_* to be contributed by Piezo1, TRPC1, and/or other channels permeable to Na^+^ and/or Ca^2+^. In contrast, TRPC1 [119] and Piezo1 [120,126,127,128] were not found to affect the light response of rods, which, in line with their location, indicates that they are not critically involved in the pressure-induced cation currents and the light- and pressure-induced opening of MSCs that we observed in the rod OS. This work reported pressure-evoked currents and analyzed current generators. In the rod OS, we identified TRPV2 and TRAAK and interpreted the pressure-evoked closure of Na^+^, Ca^2+^-permeable cation channels by the closure of TRPV2 because of its location and permeability, and if similar channels were present in the cytoplasmic membrane of the rod OS, they should have been opened by the pressure. Meanwhile, 2APB shows the effect on the light response and membrane potential of rods, which, consistent with immunological results, supports the expression of TRPV2 in rods. Although other channels like TRPV1 and TRPV3 are also responsive to 2APB [129], they have not been reported in photoreceptors. RGCs express TRPV1 [14], while a potential effect of 2APB on RGCs is less likely to affect the light response of photoreceptors. In addition to MSCs, photoreceptors also express other electrical current generators that are mechanical insensitive [130]. Due to the co-expression of multiple types of ion channels in individual cells and the limitation of MSC modulators, further studies are required to fully characterize the activity of individual MSCs in rods and cones.

## Figures and Tables

**Table 1:** Mechanosensitive channels in the outer retina.

Channel	Location
TRAAK	Photoreceptor inner segments, INL (ACs, BCs), mouse [41]
TRAAK	INL, RGL, Müller, mouse [98]
BK	Rod terminals, salamander [38]
BK	Rods, salamander [39]
BK	Photoreceptors, goldfish [40]
TRPV2 (VRL1)	Photoreceptor axon terminals, IPL, and OPL in rat, cat, primate [37]
TRPV2	Photoreceptor axons, OPL, mouse [36]
TRPV4	OPL, IPL, Müller, mouse [34]
TRPV4	OPL, IPL, pig [35]
TRPV4	OPL, IPL, BCs, RGCs, Müller, primate [16]

Note: INL: inner nuclear layer; AC: amacrine cell; BC: bipolar cell; GCL: ganglion cell layer; OPL: outer plexiform layer; IPL: inner plexiform layer.

## Data Availability

Data is contained within the article.
